# Development of a "Scissor-Tip-Separator" for adjustment of scissor blade separation and prevention of scissor blade damage during steam sterilization

**DOI:** 10.1186/s13037-022-00338-5

**Published:** 2022-08-23

**Authors:** Natthacha Chiannilkulchai, Peinjit Bhumisirikul

**Affiliations:** 1 Ramathibodi School of Nursing, Faculty of Medicine Ramathibodi Hospital, Mahidol University, 270 Rama 6 Road, Phayathai, Ratchathewi, Bangkok, 10400 Thailand; 2 Division of Perioperative Nursing, Department of Nursing, Faculty of Medicine Ramathibodi Hospital, Mahidol University, 270 Rama 6 Road, Phayathai, Ratchathewi, Bangkok, 10400, Thailand

**Keywords:** Scissor tip protector, Sharp protector, Sterilization, Surgical instruments, Tip protector

## Abstract

**Background:**

Reprocess reusable surgical instruments during steam sterilization; damage occurs to sharp scissor blades in close position, so steam cannot reach the blades. Surgical instruments' management requires standards to ensure patient safety and prevent harmful pathogens, especially in the COVID-19 pandemic. Although various devices can separate scissor blades, they do not prevent damage to cutting edges. To address the above problem, we developed a new scissor protector, the "Scissor-Tip-Separator," and evaluated its efficacy.

**Methods:**

The "Scissor-Tip-Separator" design follows the steam sterilization guideline that instrument tips must be separated. The locking handles and V groove mechanism keep the scissor blades separated while preventing damage to the cutting edges. For efficacy assessment, purposive sampling was performed to select 44 Thai perioperative nurses at Ramathibodi Hospital, Bangkok, Thailand, to evaluate the "Scissor-Tip-Separators" in 450 sterile instrument containers. All participants evaluated surgical scissors placed in the "Scissor-Tip-Separators" during instrument setup, following a problem record checklist. At the end of the fifth use, participants were asked to complete the "Scissor-Tip-Separator" Effectiveness Scale, which was used to test the structural design of the "Scissor-Tip-Separator" in terms of function, usability, and safety. The Adenosine Triphosphate surface test was also used to validate the "Scissor-Tip-Separator" cleanliness. Data were collected from August 2020 to November 2020, then analyzed via descriptive statistics.

**Results:**

The "Scissor-Tip-Separator" met the cleaning validation criteria, and in 44 uses, the physical property remained the same. The scissor shank was discovered loose from the handle before it had been unlocked (0.2–0.4%) at the 45^th^ use. Based on participants' opinions, the overall instrument effectiveness was high in terms of function, usability, and safety.

**Conclusion:**

The "Scissor-Tip-Separator" regulates scissor blade separation under sterilization guidelines; it prevents damage to cutting edges, thus ensuring patient safety. It protects against losses in a sterile field and can prevent hand injuries.

## Background

The global coronavirus disease 2019 (COVID-19) pandemic has placed many patients at high mortality risk. The patients undergoing surgeries are attacked by COVID-19 transmission; thus, elective surgeries have been canceled or postponed to conserve beds and prevent the rapid spread of COVID-19 [1–3]. However, emergency patients with and without COVID-19 require emergency surgery [2, 3]. Therefore, the management of surgical patients requires standard prevention and control practices to ensure patient and staff safety while confirming the safe use of reusable surgical instruments in anticipation of patients with COVID-19 [1, 4, 5]. The virus that causes COVID-19, severe respiratory syndrome coronavirus 2 (SARS-CoV-2), can spread via droplets, aerosols, and surfaces [1, 6]. SARS-CoV-2 is more stable on stainless steel, with an estimated median half-life of 5.6 h; it can survive on stainless steel for 4–28 days [5–8]. Most reusable surgical instruments are stainless steel, requiring steam sterilization treatment to eliminate all microorganisms and ensure patient safety for further procedures [9, 10]. The standard protocol for steam sterilization involves ensuring that steam can reach all surfaces of all instruments [11, 12]. Therefore, any interruption in the instrument management pathway can result in a high risk of infection; using unsterilized instruments can result in the transmission of harmful pathogens [10]. Reprocess reusable stainless-steel surgical instruments with cutting edges (e.g., surgical scissors) for standard requirements of steam sterilization; AORN and ANSI/AAMI ST90 have recommended holding scissor blades separation to ensure steam contact all surfaces and protecting cutting edges from damage with tip protectors [13, 14].

Surgical scissors are characterized by a lap joint with ringed handles and sharp blades. The devices such as stringers or racks can maintain instruments in the open position and allow steam contact with all surfaces [12, 14, 15]. However, these devices cannot prevent scissor blade damage because of improper containment and compression by other instruments [16, 17]. Our facility and most medical institutes in Thailand use rubber tubing to cover and prevent cutting-edge damage before steam sterilization. However, when rubber tubing covers each blade, there is sticky because of the heat interaction [12]. Moreover, insufficient space because rubber tubing fits tight so that it cannot indicate steam reaches all sharp surfaces [12]. Therefore, this solution may not meet the standard requirements for steam sterilization of surgical instruments, particularly during the COVID-19 pandemic. In addition, low-quality instruments can disrupt surgical procedures, causing delays in surgery and reduced patient safety [18, 19].

From a literature search, we identified three main features of devices used to protect surgical scissors and regulate sharp blade separation for steam sterilization. First, tip protectors include one or two caps with different shapes (e.g., round, flat, or tapered) [20]. These tip cover caps have drilled holes for steam to pass overall sharp surfaces. However, the caps are small and risk accidental loss in the surgical field [17]. Second, instrument protectors with anti-locking flaps can keep scissor blades separated, thus allowing steam to reach all surfaces. These protectors are paper-based and have a single-use feature [21, 22]. However, other heavy instruments can destroy and compress the scissor blades placed in a paper-based protector, in which the material cover has a minimum thickness of less than 2 mm [23]. Third, a puncture-resistant container is a specific tray with a rack to lock scissors in place [11]. Such a container is costly and impractical to separate scissors for protecting sharp edges in many specific containers where the scissors should aggregate into a basic instrument tray to optimize the usage and reduce costs [9, 24]. The problems with scissor protectors constitute a gap between standard guidelines and clinical practice [23, 24]. Therefore, to address the safety of reusable scissors concerning pathogens (e.g., SARS-CoV-2) and improve the quality management of reusable scissors with sharp blades, there is a need to develop a device to ensure scissors blades remain separate while preventing damage to the cutting edges. Here, we developed the "Scissor-Tip-Separator" to ensure scissor blade separation and to avoid damage to cutting edges during steam sterilization; we evaluated the effectiveness of this device.

## Methods

A design and development research process was conducted to develop the "Scissor-Tip-Separator" and evaluate its effectiveness. This overall approach is commonly used to guide the development of medical devices [25]. The details of each phase are described below.

### Design and development

After analyses of problems with surgical scissors during sterilization, we found that the scissor blades were close and had damage to the cutting edges. The device used to hold the scissor blades separate for steam sterilization and prevent damage to cutting edges had limitations, including small size, unsafe grip, risk of injury during scissor blade insertion, and risk of loss in the surgical field [13, 17]. Furthermore, some types of paper can regulate the separation of scissor blades, but they cannot prevent damage to cutting edges caused by heavy instruments. Therefore, a device was needed to ensure sharp blades remained separate while avoiding damage to cutting edges.

**Fig. 1:**
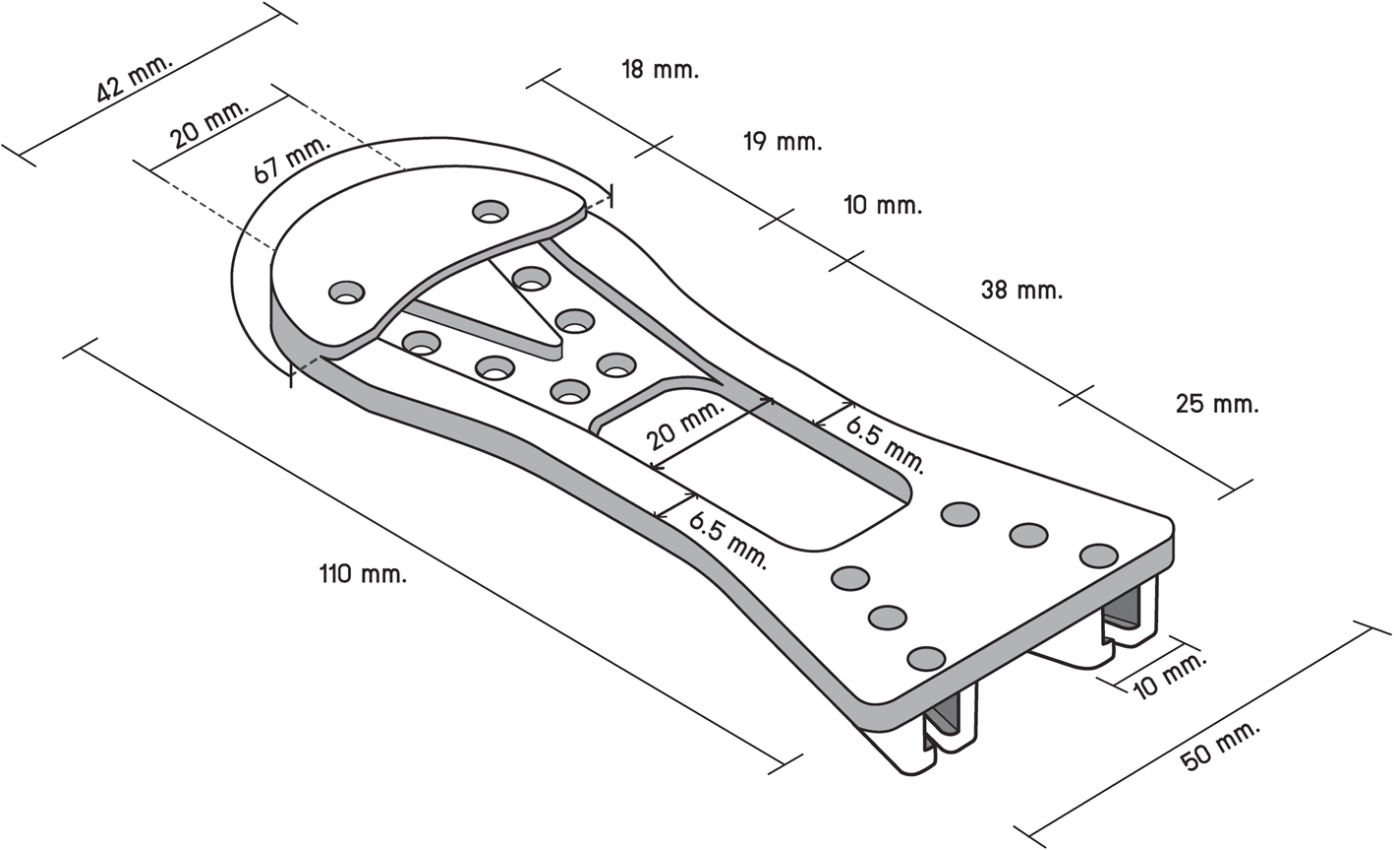
Initial version of the "Scissor-Tip-Separator" (front view)

**Fig. 2:**
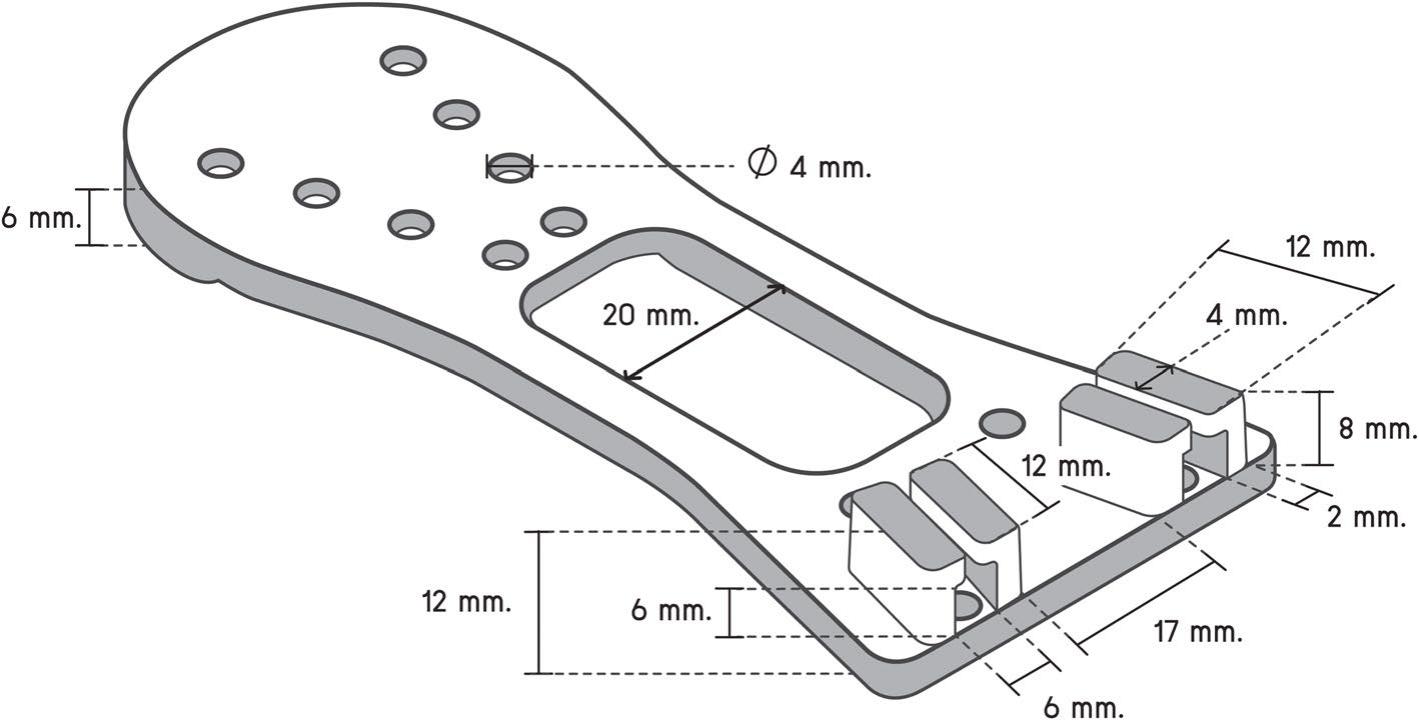
Initial version of the "Scissor-Tip-Separator" (back view)

We designed the "Scissor-Tip-Separator" following the steam sterilization guidelines that steam must reach sharp instruments at the point of use while avoiding damage to cutting edges [12, 14, 23]. We met with members of a manufacturing firm to draft the initial version of the "Scissor-Tip-Separator." We designed the "Scissor-Tip-Separator" with a reverse U-shape and chose medical silicone [26] to construct the "Scissor-Tip-Separator"; this material can be handled aseptically under high steam pressure. In the front, the width between the U-legs is 50 mm. The arc at the U-shaped dome is 67 mm. Thus, the "Scissor-Tip-Separator" length from the dome to the U legs is 110 mm. From the middle of the width up to 25 mm, it has a rectangle box of 20 × 38 mm, and the rim of both edges is 6.5 mm. The length between V legs is a curve line of 20 mm, and the height of the V-shaped is 37 mm. The V groove that ensures scissor blades are in place is 10 mm wide. The upper layer of the safeguard is 2 mm thick and 18 mm high from the middle of the lower part to the upper dome of the curved line (Fig. 1). In the back, the width is composed of two locking handles. One locking handle comprises two locking arms. Each locking arm is 4 mm wide, 8 mm high, and 2 mm thick. The length between the two locking arms is 6 mm; the distance between the two locking handles is 17 mm. The "Scissor-Tip-Separator" has 16 drill holes with a diameter of 4 mm (Fig. 2).

We evaluated the initial version of the device with ten perioperative nurses from other surgical departments at Ramathibodi Hospital; these nurses were excluded from the principal analysis. After this pilot test, one participant reported that the scissor blades were spread too narrow because the length between V legs was insufficient. Therefore, the distance between V legs was adjusted from 20 to 24 mm. Additionally, two participants reported that the scissor shanks were unlocked from the locking handle because the upper space between the two locking arms was too wide. Therefore, we redesigned the locking handle structure by adjusting the upper cavity (between locking arms) from 6 to 5 mm, the locking arm thickness from 2 to 3 mm, and the outside of each locking arm from 12 to 13 mm for bending the angle of locking arm inside. One participant suggested that although the size was sufficient to fit all five scissors, it was too big for the 5-inch Metzenbaum scissors. Therefore, we developed another size of "Scissor-Tip-Separator." The original model is large and increases the medium with a length of 87 mm.

### Implementation and efficacy evaluation

We evaluated the Scissor-Tip-Separator's effectiveness in terms of patient safety. The evaluation process included validated cleanliness of the "Scissor-Tip-Separator" before steam sterilization, documentation of problems with the "Scissor-Tip-Separator," and an assessment of participants' opinions regarding using the "Scissor-Tip-Separator."

#### Sample and setting

Our study sample comprised perioperative nurses working in the surgical department of the Main Building, Ramathibodi Hospital, Bangkok, Thailand; these nurses were selected by purposive sampling. This hospital was chosen because all surgical instrument sets were steam sterilized in standard containers suitable for assessing "Scissor-Tip-Separator" effectiveness. This study included all participants with one year of experience as perioperative nurses who evaluated the "Scissor-Tip-Separator" in the Minor Set five times during surgical instrument setup. Participants who did not meet these criteria were excluded. In total, 46 prospective nurses were identified. Forty-four perioperative nurses met the inclusion criteria and were willing to participate in the study.

#### Instruments

1. The first author developed the "Scissor-Tip-Separator Problem Record." The record consisted of nine items that were used to assess whether the structural design of the "Scissor-Tip-Separator" was working. An example item was "The scissor shank is difficult to release from the locking handle"; participants were asked to record "yes" (if they met this problem) or "no" (if they did not meet this problem). The "Scissor-Tip-Separator Problem Record" was reviewed and validated by three experts: two head nurses from two perioperative units and one infectious disease nursing instructor.
2. The first author developed the "Scissor-Tip-Separator Effectiveness Scale." The scale focused on nurses' opinions regarding using the "Scissor-Tip-Separator." The Effectiveness Scale contains 11 items within three primary subscales: "Scissor-Tip-Separator" functions, "Scissor-Tip-Separator" usability, and "Scissor-Tip-Separator" safety. Each item was answered on a five-point Likert scale, ranging from 1 (very ineffective) to 5 (very effective); higher scores indicated high effectiveness. A panel of three experts assessed the instrument's content validity; the S-CVI for the 11 items was 0.94, and CVI was 0.82. The internal consistency reliability (Cronbach's alpha) based on the opinions of the ten pilot perioperative nurses was 0.95. After assessment by all 44 perioperative nurses, the overall Cronbach's alpha coefficient for the instrument was 0.95. Cronbach's alpha was used to determine inter-item correlations among the three subscales [27, 28]. The subscales showed a Cronbach's alpha of 0.85 for the function subscale, 0.91 for the usability subscale, and 0.83 for the safety subscale. Therefore, these three subscales were analyzed as interval scales [29]. The three cut-point scores for the five-point Likert scale were as follows: (Maximum – Minimum) / Group = (5–1)/3 = 1.33. A mean score of 1.00 to 2.33 indicated low effectiveness, 2.34 to 3.66 indicated moderate effectiveness, and 3.67 to 5 indicated high effectiveness [28].
3. The Adenosine Triphosphate (ATP) Surface Test was chosen to validate the cleanliness of the reusable surgical instruments. Although several methods are used to verify cleaning efficacy, they are not currently regulated by the FDA [30]. The study of Veiga-Malta suggested that the ATP method is more useful in the central sterile supply department (CSSD) because of its practicality [31]. In addition, our CSSD used ATP as a standard method, and staff in CSSD who have been trained adequately in the use of the ATP technique. Therefore, the authors used the ATP method to evaluate surgical instruments' cleanliness. The ATP Surface Test consists of a swab for the surface test and the ATP testing solution that measures with a luminometer. The reaction between ATP testing solution and biological residues contributed to the light signal measured in relative light units (RLUs). The ATP level is associated with organic residue contamination [32, 33]. Therefore, the cut-off cleanliness level was set at 150 RLUs. The ATP level < 150 RLUs indicates 'clean' or passing cleaning criteria. While the ATP level > 150 RLUs indicates 'dirty' or failing cleaning criteria and should reprocess cleaning before being sent to sterilization [33].

#### Procedure for preparation of reusable surgical scissors

The instrument sets used to evaluate the "Scissor-Tip-Separator" were designated "Minor Sets." This study used 10 Minor Sets labeled with numbers 1 to 10. We trained the sterilization staff to prepare "Scissor-Tip-Separators" and pack them in paper packages for steam sterilization of the Minor Set. The following process was used to prepare "Scissor-Tip-Separators" in the Minor Set for steam sterilization.

**Fig. 3:**
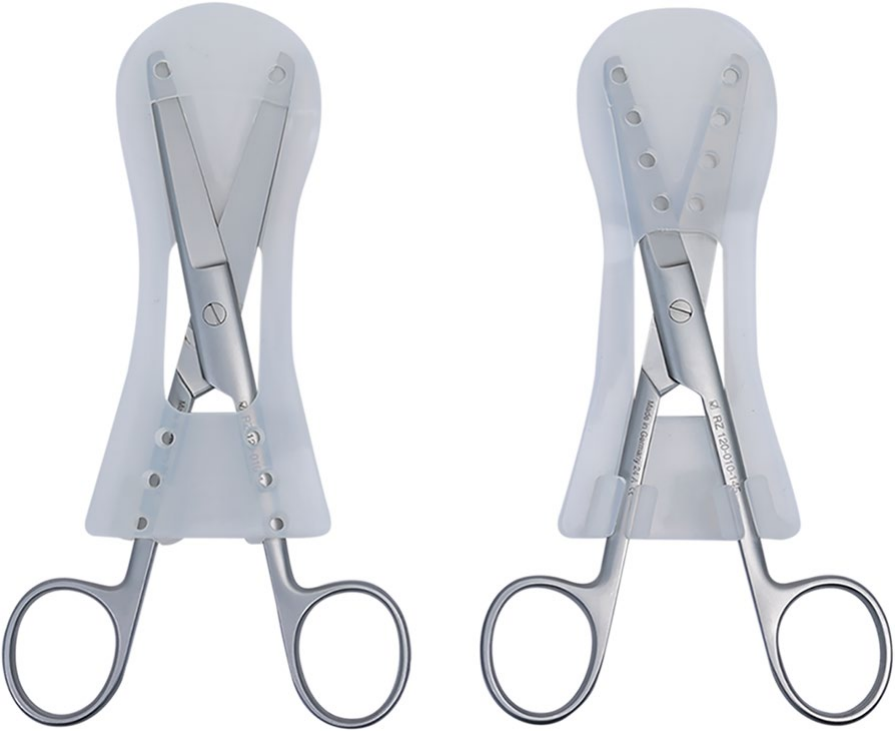
The model of the "Scissor-Tip-Separator"

1. Each Minor Set comprised three types and five pieces of surgical scissors: one Mayo, two Metzenbaum, and two Suture scissors. Therefore, we used five "Scissor-Tip-Separators": two medium sizes for the two sizes of Metzenbaum scissors and three large sizes for Mayo and Suture scissors. The sterilization staff began preparation by inserting scissor blades through the rectangular box at the back into the front; the staff placed scissor blades into the V-groove, with the sharp tips in the safeguard. The staff then pressed the scissor shanks into the locking handles at the back (Fig. 3).
2. Subsequently, the sterilization staff placed five Class IV steam chemical indicators (integrating indicators) in the "Scissor-Tip-Separator" to evaluate whether steam could reach the entire scissor blade surface [12], then wrapped the prepared "Scissor-Tip-Separators" in the sterile paper. Finally, the Minor Sets were subjected to steam sterilization.

#### Data collection

Each day, one research team member checked the operation schedule that used a Minor Set and met the participants who were the scrub nurses or circulating nurses in the minor surgeries. The researcher explained the activity's objective and asked the participants for cooperation. The process began with a demonstration of the "Scissor-Tip-Separator" release. Next, participants were asked to examine and verify the scissor package when they opened the Minor Set, following the checklist in the problem record. Then, the five indicators were checked and evaluated for color, from the rejection zone to the acceptance zone. If the black color did not reach the acceptance zone, "yes" was marked in the "Problem Found" item. At the end of the surgical procedure, the "Scissor-Tip-Separators" were sent to clean in the washing machine at the CSSD and subjected to validate the cleanliness of the reused medical device with the ATP Test; they were then placed in a sterile package for steam sterilization. The cleaning test began with the staff of CSSD randomly selecting one of 30 large "Scissor-Tip-Separators," and one of 20 medium "Scissor-Tip-Separators" from 10 Minor Sets. Two sizes of "Scissor-Tip-Separators were subjected to the ATP Test once daily for 45 days and recorded the ATP levels for two sizes each day. In the operating room, the scrub and circulating nurses checked the "Scissor-Tip-Separators" in each Minor Set, following the problem record checklist for a total of 450 sets (i.e., 45 days × 10 sets/day). At the end of the surgical procedure, the names of the perioperative nurses who checked the "Scissor-Tip-Separators" were recorded. Participants who checked the "Scissor-Tip-Separator" in Minor Set five times (i.e., after five surgeries) were asked to complete an effectiveness evaluation scale within 15–20 min. These data were collected from August 2020 to November 2020.

#### Data analysis

Descriptive statistics, including frequencies and percentages, were used to analyze the ATP level and identify problems using the "Scissor-Tip-Separator." The "Scissor-Tip-Separator Effectiveness Scale" findings were analyzed in two parts. First, the overall items and the subscales regarded as an interval scale were analyzed as a group via means and standard deviations [27, 28]. Second, individual items considered an ordinary scale were analyzed using medians and frequencies to measure central tendency and dispersion [28].

## Results

From August 2020 to November 2020, all 44 participants used the "Scissor-Tip-Separator" in Minor Sets 450 times (i.e., 450 surgeries). The participants checked 2250 chemical indicators, all turning back to the accepted area. Evaluate the "Scissor-Tip-Separator" cleanliness 45 times, ATP level for large size range 4–29 RLUs mean 16.44 SD 5.29 and for medium size range 3–58 RLUs mean 17.40 SD 9.67.

The problem record showed that two samples of 450 surgeries presented the scissor shanks were released from the locking handle of the large "Scissor-Tip-Separator" before unlocking (0.4%) and one of 450 times lost from the medium size (0.2%). The physical properties of the "Scissor-Tip-Separator" did not change during 44 uses. For the 45^th^ use, the locking handle of the large "Scissor-Tip-Separator" (sets 4 and 10) and medium "Scissor-Tip-Separator" (set 9) were slightly changed, as shown in Table 1.

**Table 1:** “Scissor-Tip-Separator” problem record (*N* = 450)

**Problems during use of the "Scissor-Tip-Separator."**	**Size**	**Occurred** **N (%)**	**Did not occur** **N (%)**	**Remarks**
1. The scissors' sharp end penetrated the "Scissor-Tip-Separator" tip hole.	Large	0	450 (100)	
	Medium	0	450 (100)	
2. The sharp end of the scissors penetrated the back of the "Scissor-Tip-Separator."	Large	0	450 (100)	
	Medium	0	450 (100)	
3. The scissor shank was loose and released itself from the locking handle before unlocking.	Large	2 (0.4)	448 (99.6)	Set 10, day 45 Set 4, day 45
	Medium	1 (0.2)	450 (99.8)	Set 9, day 45
4. The scissor shank was difficult to release from the locking handle	Large	0	450 (100)	
	Medium	0	450 (100)	
5. The scissor tip slipped out of the safeguard because the rectangular box in the middle was too wide.	Large	0	450 (100)	
	Medium	0	450 (100)	
6. Water was contained in the V grooves and at the safeguard	Large	0	450 (100)	
	Medium	0	450 (100)	
7. Indicators inserted between the scissors and the "Scissor-Tip-Separator" did not change color or turn black, or the black color did not reach the acceptance zone.	Large	0	450 (100)	
	Medium	0	450 (100)	
8. The scissor blades were deformed or damaged upon release from the "Scissor-Tip-Separator."	Large	0	450 (100)	
	Medium	0	450 (100)	
9. The "Scissor-Tip-Separator" was broken or adhered to the scissors or exhibited changes in physical characteristics.	Large	2 (0.4)	448 (99.6)	Set 10, day 45 Set 4, day 45
	Medium	1 (0.2)	450 (99.8)	Set 9, day 45

*Abbreviations*: *N* Number of surgeries

The mean score of all items evaluated was 4.85 ± 0.30 (Table 2). Mean scores > 4.8 points were computed from the analysis of all three subscales. The subscale scores were 4.88 ± 0.29 for function, 4.83 ± 0.33 for usability, and 4.83 ± 0.33 for safety. Most nurses (*n* = 36, 81.8%) gave all items a score of 5 (median = 5 points).

**Table 2:** Participants’ opinions regarding using the "Scissor-Tip-Separator" (*N* = 44)

**Evaluation items**	**Min**	**Max**	**Median**	**M ± SD**	**Level of Effectiveness**
**1. The function of the "Scissor-Tip-Separator."**				**4.88 ± 0.29**	**High**
1.1 The "Scissor-Tip-Separator" is a device that regulates scissor blade separation and prevents scissor blades from returning to their normal position	4	5	40 (90.9)		
1.2 The "Scissor-Tip-Separator" holds scissor blades to allow steam to reach all surfaces; this can be confirmed by observing that the indicator inserted in the "Scissor-Tip-Separator" turns black in the acceptance zone	4	5	38 (86.4)		
1.3 The "Scissor-Tip-Separator" tip guard protects the scissors' sharp blades from chipping, bending, and deformation	4	5	38(86.4)		
**2. Usability of the "Scissor-Tip-Separator"**				**4.83 ± 0.33**	**High**
2.1 The V groove fits the scissor blades; it is compatible with different types and sizes of scissors	4	5	36 (81.8)		
2.2 The rectangular box in the middle is appropriate for inserting the scissors from the back toward the front; scissors are easily inserted into the safeguard	4	5	36 (81.8)		
2.3 The scissor shanks can easily be pressed and released from the locking handle. Simultaneously, the "Scissor-Tip-Separator" firmly holds the scissor shanks in the locking handle	4	5	37 (84.1)		
2.4 The U shape of this device makes it easy to grip; it does not slip when surgical scissors are removed from the "Scissor-Tip-Separator."	4	5	37 (84.1)		
2.5 The safeguard and drilled holes allow steam to reach all scissor surfaces easily	4	5	36 (81.8)		
**3. Safety of the "Scissor-Tip-Separator"**				**4.83 ± 0.33**	**High**
3.1 After steam sterilization, the "Scissor-Tip-Separator" was not melted or adhered to the scissors	4	5	37 (84.1)		
3.2 The "Scissor-Tip-Separator" can be sterilized multiple times under high steam pressure without changing its form or deteriorating	4	5	36 (81.8)		
3.3 The "Scissor-Tip-Separator" can be used to pack scissors for sterilization or release scissors for surgical procedures without injury	4	5	37 (84.1)		
**Total**				**4.85 ± 0.30**	**High**

*Abbreviations*: *N* Number of participants, *Min* Minimum score, *Max* Maximum score, *Median* Median score, *M* Mean, *SD* Standard deviation, *n* Number of participants who rated the item with the median score; 1, very ineffective; 2, ineffective; 3, neither ineffective nor effective; 4, effective; 5, very effective

## Discussion

This study was conducted to develop the "Scissor-Tip-Separator" to standardize the separation of scissor blades and prevent damage to cutting edges. Our results showed that the "Scissor-Tip-Separator" could effectively maintain scissor blade separation and avoid damage to cutting edges, and it passed the validation of the cleaning process.

### Cleaning valid surgical instruments reprocessing

ATP is one of many methods used to verify cleaning conditions [30, 34]. The results showed that the ATP levels were 16.44 ± 5.29 RLUs for the large "Scissor-Tip-Separator" and 17.40 ± 9.67 RLUs for the medium "Scissor-Tip-Separator," indicating they met the cleanliness criteria for standard reprocessing reusable surgical instruments in pre-sterilization (< 150 RLUs) [33]. Additionally, ATP levels less than 100 RLUs indicate lower biological residue contamination [35, 36]. Following standard practices for surgical instrument cleaning and care, the AORN and AAMI recommend the implementation of cleaning test procedures to ensure that instruments/devices are cleaned effectively [12, 14]. Validating the cleanliness of reusable instruments is essential in reprocessing sterilization [37]. If cleaning is inadequate, biological contamination can form a thin layer of microorganisms, which acts as a physical barrier to prevent steam from reaching the surface of the devices [33, 38, 39]. Inadequate and failed cleaning can reduce the effectiveness of sterilization, threatening patient health and potentially leading to infection outbreaks in surgical departments [34, 40].

### Function

The problem record showed that all 2250 chemical indicators inserted between the scissors and the "Scissor-Tip-Separators" turned black from the rejection zone to the acceptance zone; no water droplets were present in the space of the V groove or the safeguard. The essential step is to ensure the proper sterilization of the scissors to the point of use; steam must be able to penetrate chemical indicators from the rejection to the acceptance zones by changing the color from white to black [41]. Thus, the scissors within the "Scissor-Tip-Separators" inside a steam sterilizer (temperature 134 C time 3 min) have sufficient space for steam to move freely, and drilled holes allow steam penetration to contact all surfaces, enhancing rapid drain and evaporation. According to the standard protocol for surgical instruments in steam sterilization, steam must reach all surfaces of all devices, then rapidly evaporate [14]. Consist with the ANSI/AAMI ST79 recommends that tip protectors be steam-permeable [12]. The sterile items that become moisture or droplets are considered insufficient space for drainage and evaporation; they may become contaminated, improper drying can lead to corrosion in the instruments, and thus fail to meet standard requirements [10, 14, 16, 42]. Biological transmissions can occur if medical devices are not reprocessed under standard guidelines [43]. Improperly resterilized surgical instruments used in many procedures may cause infections in numerous patients [38]. Therefore, our study showed that steam (temperature 134 C time 3 min) could penetrate all surfaces of the scissor blade packed in the "Scissor-Tip-Separator" that is proper to destroy microorganisms, including SARS-CoV-2 [44].

Historically, in an annual year, our surgery department records that there are damaged scissors that require repair of 36 pieces and outright replacement of ten pieces [unpublished record]. However, since using the "Scissor-Tip-Separator" to protect scissors for four months, the problem record showed no reports of scissor blade deformation or damage upon removal of the "Scissor-Tip-Separator." Confirm with, 38 participants (86.4%) reported a score of 5 points for the "Scissor-Tip-Separator" to protect scissor blades (at the point of use) from chipping, bending, and deformation. This result can be attributed to the 10 mm width of the V groove, which is enough to hold the scissor blades in place. According to ANSI/AAMI ST79, sharp items should be protected from damage; tip protectors should loosely fit [12]. Even though there are options for testing scissor performance in the current situation [13], our surgical department obtained scissor performance from surgeon complaints during surgery, including unsharpness, loss of alignment, and loose pivot joint. After using "Scissor-Tip-Separators" to protect scissors, 11 perioperative nurses reported additional suggestions that the surgeons did not complain or require replacing the scissors during surgery. In addition, the scrub nurses could quickly inspect and check the scissors' performance during surgical setup. Damage at the point of use renders instruments unusable, resulting in surgical delays that negatively impact patient safety and increase staff workload by requiring the preparation of new instruments [16, 19]. Poor quality control of surgical instruments leads to low-quality instruments [19]. Any general increase in operating room costs has substantial socio-economic impacts nationwide [18].

The total cost of the "Scissor-Tip-Separators" that were used on 10 Minor sets was 15,000 baht or 420 USD ("Scissor-Tip-Separator" 300 baht/piece, five scissors/set, and used 10 Minor sets = 15,000 baht) compared to use the repair and replacement scissors which cost 44,760 baht or 1252 USD (repair 160 baht/piece, replace 6 Metzenbaum 6,000 baht/piece and 4 Suture scissors 2,500 baht/piece = 44,760 baht). Thus, using the "Scissor-Tip-Separator" protect scissors can reduce our institute's cost of 832 USD.

### Usability

In the usability assessment of the "Scissor-Tip-Separators," there were no reports of the scissor tips slipping out of the safeguards. Consistent with this result, participants reported that the "Scissor-Tip-Separators" had high usability. These findings are presumably because the rectangular box in the middle is 20 × 38 mm, which is the proper size for maintaining the pivot joint in place, keeping the scissor blades in the V groove, and holding the scissor tips in the safeguard. The V groove locks the scissor blades into the safeguard functions as a locking system. According to the steam sterilization protocol of sharp instruments, keep them orderly, do not touch their points, and prevent damage with perforated tip protectors [12, 14]. Thus, the V groove and safeguard function as perforated tip protectors, protecting the scissor tips in the safeguard.

Concerning the locking design, no difficulty was reported by scrub nurses in releasing the scissor shanks from the locking handles. Consistent with this result, 84.1% of participants reported that "the scissor shanks were easily pressed and released from the locking handles, while the locking handles were secure and prevented the scissor shanks from slipping out." Thus, the locking handles function as a secondary locking system for ensuring that scissor blades remain separate. The "Scissor-Tip-Separator" has a U shape; when releasing or placing surgical scissors, the "Scissor-Tip-Separator" can be firmly gripped to prevent slippage. Poor structural design, such as difficulty in use, may increase patient safety risks, as mentioned in the assessment of medical device usability [45]. The dual locking systems of the "Scissor-Tip-Separator" allow steam to reach all scissor blade surfaces and prevent damage to cutting edges; these are considerably different from other available tip protectors.

### Safety

The safety assessment revealed no instances in which the scissor tips penetrated out of the safeguards or exited from the back of the "Scissor-Tip-Separators." Consistent with these findings, participants' safety ratings showed high effectiveness. The V shape, which is 37 mm in length, firmly locks the scissor blades (28–34 mm distance from the bottom to the middle part of the V legs) in place and protects scissor tips from penetrating out of the safeguard, thereby preventing user injury. In addition, the safeguard, which is 18 mm in length and 2 mm in thickness, firmly protects against scissor blade damage by other instruments' compression [23]. Thus, the V shape and the safeguard successfully prevent user injury during pre- sterilization and instrument setup. The size of the "Scissor-Tip-Separator" may have influenced these positive findings, such that the "Scissor-Tip-Separator" is easy to hold and protect from loss in the surgical field. Surgeons in operating rooms expect to have all instruments available in terms of type and quality [18]. If scissor blades are damaged, there is a risk of tissue damage and surgical delays.

For evaluating the physical properties, the "Scissor-Tip-Separators" did not exhibit breakage or adhesion to the scissors; they were consistent; however, the locking handles were slightly dilated in the 45^th^ round of sterilization. These findings were consistent with participant ratings, indicating that the materials used to produce the "Scissor-Tip-Separator" did not melt or adhere to scissors after steam sterilization. The "Scissor-Tip-Separator" can be resterilized under high steam pressure without changing its form or deteriorating. The explanation is that the "Scissor-Tip-Separator" made of medical-grade silicone is resistant to high temperatures (> 200 °C); it does not lose physical characteristics or exhibit substantial degradation [26]. Therefore, this silicone is a material that is appropriate for producing the "Scissor-Tip-Separator" and safe for steam sterilization.

### Limitations

There may be several limitations in this study. First, we packed surgical scissors placed in the "Scissor-Tip-Separator separated from other instruments. To decrease bias, we packed the scissors together, similar to those in Minor Set. Second, our study used purposive sampling, which involved a small sample size in assessing participants' opinions. However, we used specific inclusion criteria and total population sampling to minimize bias related to sample selection [46]. Third, although our participant sample size was small, our primary data comprised 450 problem record checklists and cleaning validation data from 450 Minor Sets; we presumed that these were sufficient data. Forth limitation, we used self-reports to assess participants' opinions regarding the "Scissor-Tip-Separator," which may have led the participants to overestimate their expertise or knowledge. However, to minimize bias, experts were included in our measurement validation process; we also conducted a pilot study before the sample group used the measurements [47]. Following limitation, we included participants from only one medical institution while assessing "Scissor-Tip-Separator" effectiveness. This approach limits generalizability to other perioperative nurses who might use the "Scissor-Tip-Separator" in different contexts. The final limitation, our study focused only on evaluating the 'Scissor-Tip-Separator,' and we did not compare it with other standard tip protectors. Therefore, further research should be conducted to compare the "Scissor-Tip-Separator" with other standard tip protectors on the cutting edge of surgical scissors.

## Conclusions

Our results showed that the locking handles and safeguard of the "Scissor-Tip-Separator" could ensure the scissor blades remained separated, allow steam to reach the entire surface of surgical scissors, and prevent damage to cutting edges. In addition, perioperative nurses can firmly release surgical scissors from the "Scissor-Tip-Separators," promoting themselves and patient safety. In this study, we used a problem record to describe "Scissor-Tip-Separator" functions and participants' opinions; this enabled the identification of possible design errors and assessment of "Scissor-Tip-Separator" effectiveness. The crucial finding revealed that the size of the scissor protector should be large enough to grip to prevent loss in the surgical area and protect against personal injury. Furthermore, the CSSD involves preparing and managing surgical instruments and should apply tip separators to surgical scissors in an appropriate size. Accordingly, we designed the "Scissor-Tip-Separators" with different sizes to match different types and sizes of surgical scissors.

## Data Availability

The datasets used and analyzed during the current study are available from the corresponding author upon reasonable request.
